# LOXL2 promotes vasculogenic mimicry and tumour aggressiveness in hepatocellular carcinoma

**DOI:** 10.1111/jcmm.14039

**Published:** 2018-12-01

**Authors:** Bing Shao, Xiulan Zhao, Tieju Liu, Yanhui Zhang, Ran Sun, Xueyi Dong, Fang Liu, Nan Zhao, Danfang Zhang, Lili Wu, Yong Wang, Meili Wang, Jie Meng, Xian Lin, Baocun Sun

**Affiliations:** ^1^ Department of Pathology Tianjin Medical University Tianjin China; ^2^ Department of Pathology Cancer Hospital of Tianjin Medical University Tianjin China; ^3^ Department of Pathology General Hospital of Tianjin Medical University Tianjin China; ^4^ Tianjin Nankai Hospital Tianjin China

**Keywords:** angiogenesis, cell polarity protein, prognosis, tumour metastasis, tumour progression

## Abstract

Lysyl oxidase‐like 2 (LOXL2) has shown to promote metastasis and poor prognosis in hepatocellular carcinoma (HCC). Also, we have previously reported that vasculogenic mimicry (VM) is associated with invasion, metastasis and poor survival in HCC patients. In the present study, we investigated molecular function of LOXL2 in HCC and VM. We used the immunohistochemical and CD31/periodic acid‐Schiff double staining to detect the relationship between LOXL2 and VM formation. We performed the gain and loss of function studies and analysed the migratory, invasion and tube formation in HCC cell lines. We analysed the function of LOXL2 in VM formation and HCC metastasis both in vitro and in vivo. We have showed that LOXL2 was overexpression in HCC and was positively correlated with tumour grade, metastasis, VM formation and poor survival in 201 HCC patients. Secondly, our studies have showed that LOXL2 overexpression in HCC cells significantly promoted migration, invasion and tube formation. Finally, we found that LOXL2 may increase SNAIL expression, thereby enabling VM. Our study indicated that LOXL2 may promote VM formation and tumour metastasis by collaborating with SNAIL in HCC. What's more, the overexpression of LOXL2 indicated a poor prognosis in HCC patients.

## 1 INTRODUCTION

Hepatocellular carcinoma (HCC) claims more than 600 000 lives every year worldwide.^1^ Its high mortality rate is mainly attributed to vascular invasion and metastasis.^2^, ^3^, ^4^ An adequate blood supply is also crucial for tumour growth and metastasis.^5^, ^6^, ^7^, ^8^ It has been taken for granted that only endothelial cells could form blood vessels. However, this opinion has been challenged by the new discovery that tumour cells also form vascular networks in melanoma. The process by which tumour cells form a vessel is called vasculogenic mimicry (VM). This concept was introduced by Maniotis et al in 1999 and describes a phenomenon through which highly aggressive uveal melanoma can form capillary‐like and extracellular matrix (ECM)‐rich tubular networks in vivo without the involvement of endothelial cells.^7^, ^9^, ^10^, ^11^, ^12^

Since then, many studies have investigated the underlying molecular mechanism of VM. VM has been accepted as a novel form of angiogenesis in the tumour microenvironment in several different types of cancers, which are all highly malignant and can easily metastasize.^6^, ^10^, ^13^, ^14^ Current studies of the mechanism of VM mainly focus on the remodelling of the ECM and tumour microenvironments.^11^, ^14^ Our previous work has shown that VM is associated with the invasive and metastatic potential of tumour cells and with poor clinical outcomes.^15^ To date, three microcirculation patterns have been identified in HCC: endothelium‐dependent vessels, VM and mosaic vessels.^10^, ^12^ However, because the tumour blood supply patterns are so complex, the effects of anti‐angiogenic therapy on HCC patients are unsatisfactory. Therefore, more research on the tumour blood supply is needed.

Lysyl oxidases constitute a family of secreted copper‐dependent amine oxidases that catalyse the oxidative deamination of the ε‐amino group in peptidyl lysine residues, thus promoting covalent protein cross‐linkages.^16^, ^17^, ^18^ Lysyl oxidase‐like 2 (LOXL2) is a member of the lysyl oxidase (LOX) family, of which five members have far been identified to date, including one LOX, and four LOX‐like proteins, LOXL1–4. LOXL2 play an important role in several processes, such as cell adhesion, cell migration and invasion, epithelial cell plasticity and the epithelial‐mesenchymal transition (EMT).^16^, ^17^, ^18^, ^19^, ^20^, ^21^ Moreover, LOXL2 plays a key role in the stabilization of collagen and elastin fibres in ECM remodelling, which plays a major role in the development of a functional vascular system.^20^, ^22^, ^23^, ^24^ Bignon et al recently showed that LOXL2 is involved in angiogenesis, which is driven by hypoxic tumour microenvironments.^25^, ^26^ LOXL2 can affect endothelial cell proliferation and migration, which are necessary for capillary formation.^23^, ^25^, ^26^, ^27^ More interestingly, accumulating evidence indicates that LOXL2 can promote invasion and metastasis in basal‐like breast cancer cells by collaborating with SNAIL and lethal giant larvae (LLGL2)^28^, ^29^; LOXL2 was also described as a prognostic marker in larynx squamous cell carcinomas.^30^ SNAIL is a transcription factor that is involved in the EMT^31^, ^32^; Our previous research data revealed that the EMT may be involved in VM in HCC.^14^, ^33^ Moreover, LLGL2, which is a component of the cell polarity complex (Scribble complex), has previously been shown to be downregulated by EMT factors such as Snail1/Snail2 and/or ZEB1.^14^, ^33^

Although the expression of LOXL2 and its implications have been shown in HCC, the correlation between LOXL2 and VM in HCC and their relevance as clinical parameters remain unclear. In this study, we attempted to identify the effects of LOXL2 on tumour VM.

## 2 MATERIALS AND METHODS

### 2.1 Patients

Tissue specimens were obtained from the Tumor Tissue Bank of the Tianjin Cancer Hospital. The specimens were from 201 patients between 2001 and 2009. The diagnoses of these HCC samples were verified by pathologists. Detailed pathological and clinical data were collected for all samples, including Edmondson tumour grade, metastasis and survival duration. Paraffin‐embedded tumour tissue samples were collected from patients who had not undergone therapy prior to the surgical operation on the tumour. The use of these tissue samples was approved by the Institutional Research Committee.

### 2.2 Immunohistochemical and double histochemical staining methods

The sections were pre‐treated with microwaves, blocked and incubated with a series of antibodies (Table S2). The staining systems used in this study were PicTure PV6000 (Zhongshan Chemical Co., Beijing, China) and Elivision Plus (Zhongshan Chemical Co.). Finally, the sections were counter‐stained with haematoxylin or periodic acid‐Schiff (PAS). Phosphate‐buffered saline was used in place of the primary antibodies for the negative control.

### 2.3 Counting methods

The results of immunohistochemical (IHC) staining were assessed through microscope by two pathologists. The assessing method was described in the supplement material. VM was identified by the presence of red blood cells in vessels lined by tumour cells, and not by endothelial cells, and by the absence of necrosis and inflammatory cells infiltrating the area surrounding the channels.

### 2.4 Cell culture and transfection

The HCC cell lines HepG2, HepG3B and SMMC7221 we used were obtained from the American Type Culture Collection (Rockville, MD, USA) in 2012 and authenticated using short tandem repeat (STR) analysis by Genewiz Inc. in 2014. STR analysis showed that the submitted samples were in good agreement with the reference cell lines. The HCC cell line Bel7402 was obtained from KeyGEN BioTECH and we finished our studies within 6 months. HepG2 cells were cultured in Modified Eagle's Medium supplemented with 10% foetal bovine serum, SMMC7721 cells were cultured in Dulbecco's Modified Eagle's Medium supplemented with 10% foetal bovine serum, while Bel7402 and HepG3B were cultured in Roswell Park Memorial Institute 1640 supplemented with 10% foetal bovine serum (Invitrogen). The vectors were transfected into cells using a percutaneous ethanol injection (cat no. 23966; Polysciences, Inc.).

### 2.5 Expression plasmids

The full‐length LOXL2 and SNAIL complementary DNAs (cDNAs) were generated from normal human embryo total cDNAs, digested with XhoI/EcoRI and subcloned into pcDNA3.1 vectors. The resulting constructs were confirmed by DNA sequencing. *SNAIL* and *LOXL2* Gene Silencing used the small interfering RNA (siRNA) kit (pGP‐Twist1‐shRNA) purchased from GeneCopoeia (US). Puromycin was used as the stable cell line selection marker.

### 2.6 RNA extraction and quantitative reverse transcription‐PCR

Total RNA was extracted using TRNzol A+ Reagent (TaKaRa Biotechnology Co., Ltd., Japan), according to the manufacturer's instructions; cDNAs were prepared using the Quantscript RT Kit (Tiangen Biotech). Quantitative PCR (qPCR) was performed with a 7500/7500 Fast Real‐Time PCR System (Applied Biosystems), Tli RNaseH Plus (RR820A; TaKaRa). Quantitative reverse transcription‐PCR (qRT‐PCR) was performed as previously described.^12^ The primers used for qRT‐PCR are listed in Table S8.

### 2.7 Western blot analysis

The whole cell lysates were separated by sodium dodecyl sulphate–polyacrylamide gel electrophoresis and transferred onto polyvinylidene difluoride membranes (Millipore). The blots were blocked and incubated with the appropriate antibody (Table S2), followed by incubation with a secondary antibody (1:2000; Santa Cruz Biotechnology). The blots were developed using an enhanced chemiluminescence detection kit (Amersham Pharmacia Biotech, Piscataway, NJ, USA). For the protein loading analyses, a monoclonal beta‐actin antibody (1:200; Santa Cruz Biotechnology) was used.

### 2.8 Immunofluorescence staining

Cells were plated onto chamber slides and fixed in ice‐cold methanol. The primary antibodies against LOXL2 and SNAIL were used at a 1:400 working dilution. Fluorescein isothiocyanate‐ and tetramethylrhodamine isothiocyanate‐conjugated mouse and rabbit immunoglobulin G antibodies (Santa Cruz Biotechnology) were used as labels for the immunofluorescence assay. After immunolabelling, the cells were washed, stained with DAPI (Sigma), mounted and then viewed with a fluorescent microscope (Nikon, Japan).

### 2.9 3D cultures

Tumour cells were transfected, incubated 24 hours and mixture‐seeded with Matrigel (Collaborative Biomedical), and the matrix was allowed to polymerize. The addition of conditioned media with 10% foetal bovine serum (Hyclone) allowed us to perform pre‐treatment and continuous treatment regimens during the 10‐day incubation period in 3D cultures. The cells were collected from the Matrigel with trypsin, to which Trizol or RIPA buffer was added to isolate the total RNA or protein from the cells.

### 2.10 Invasion and wound healing assay

The cell migration assay was performed with Transwell cell culture inserts (Invitrogen). The transfected cells were maintained for 48 hours and allowed to migrate for another 24 hours. The migrated cells were stained with a crystal violet solution and its absorbance was determined at 595 nm. In the wound healing assays, cell motility was assessed by measuring the movement of cells into a scrape. The speed of wound closure was monitored after 12 and 24 hours by measuring the ratio of the distance of the wound at 0 hours. Migration/invasion assays were performed as reported.^34^ Each experiment was also performed in triplicate.

### 2.11 Murine xenograft model

The orthotopic transplantation tumour model and the HCC metastatic model was made by injecting 5 × 10^6^ HCC cell into the 4‐ to 6‐week‐old nude mice. Then the mice were monitored for 4‐5 weeks and tumour sizes were measured daily using a caliper. After the observations were complete, the mice were killed, and then used for the sequent histological examination. The detail method was described in the supporting material.

### 2.12 Statistical analysis

In this study, we evaluated all data using SPSS 17.0. All *P*‐values were two‐sided, and *P* < 0.05 was considered significant. The significant groups are marked with an asterisk in the figures. Pearson's chi‐squared test was then used to compare the differences in protein expression between the metastatic and non‐metastatic groups. Moreover, forward selection of the stepwise discriminant analysis was then used to establish a combination formula to predict the metastatic potential of the second set of HCC samples. Pearson's chi‐squared test was then used to compare the difference between the predicted and actual results and to validate the predictive value of the differentially expressed proteins. For the survival analysis, survival curves were produced with the Kaplan–Meier method. Differences in the survival curves were assessed by the log rank test.

## 3 RESULTS

### 3.1 LOXL2 cytoplasm expression was significantly correlated with VM in HCC and its upregulation is associated with poor patient prognosis

**Figure 1:**
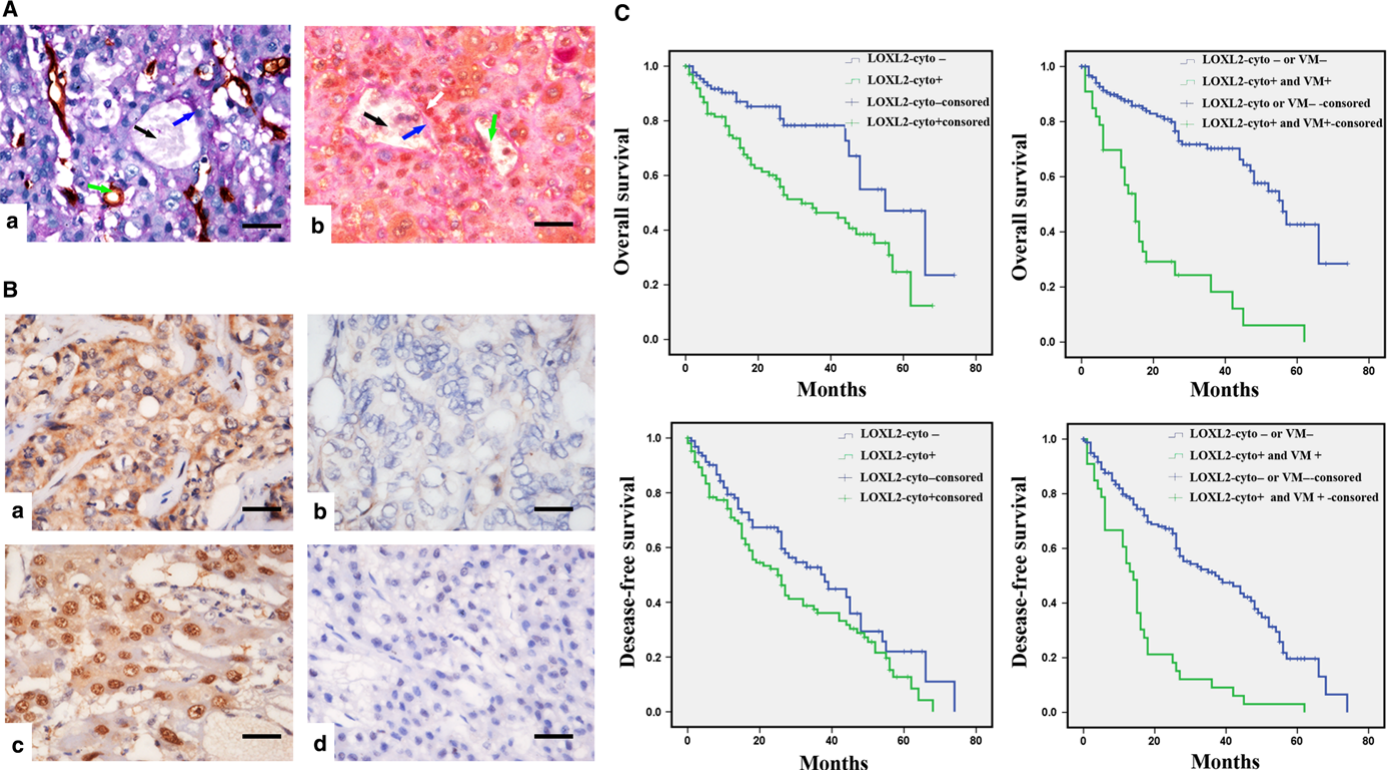
The correlation between LOXL2 cytoplasmic expression with VM and poor prognosis in clinical samples. (A) Evidence of VM in HCC and the results of co‐staining of LOXL2 and VM. (i) Evidence of VM in HCC. Vasculogenic mimicry is present in the HCC tissue, as shown by CD31/PAS double staining. Blue arrow: The double‐stained cells are CD31^−^/PAS ^+^ cells, which indicate that they are from the VM tissues formed by the tumour cells. Black arrow: the cells are red blood cells. Green arrow: The cells are CD31^+^, indicating that they were from endothelium‐dependent vessels (a). (ii) The results of co‐staining of LOXL2 and VM. The results show that the tissue which has VM formation, usually expresses high level of LOXL2. In the co‐staining of LOXL2 and VM, White arrow: brown yellow indicate the positive for LOXL2 staining, and atropurpureus indicate the positive for CD 31 staining. Blue arrow: The double‐stained cells are CD31−/PAS+ cells, which indicate that they are from the VM tissues formed by the tumour cells. Black arrow: the cells are red blood cells. Green arrow: The cells are CD31+, indicating that they were from endothelium‐dependent vessels (b). (B) The relationship between LOXL2 and VM formation. The immunohistochemical staining for LOXL2 cytoplasmic expression in VM‐ and VM+ HCC samples (a, b); the immunohistochemical staining for LOXL2 nuclear expression in VM‐ and VM+ HCC samples (c, d). (C) Patients with VM and LOXL2‐c expression had a shorter survival period than those without VM or LOXL2‐c expression. The left two panels showed that patients with LOXL2‐c expression had a shorter survival period than those without LOXL2‐c expression; the right two panels showed that patients with both VM formation and LOXL2‐c expression had a shorter survival period than those without VM formation or LOXL2‐c expression

To assess the relationship between LOXL2 and VM in HCC, we first performed IHC staining on 201 HCC tissue sections. The results showed that LOXL2 is located in both the nuclei and in the cytoplasm of HCC cells. We assessed both the intensity and the percentage of positive cells using previously described criteria (see Section 2 or Supplement Material). Then, using CD31/PAS double staining, vascular‐like patterns, which were formed by HCC cells and contained red blood cells and CD31^−^/PAS^+^ cells, lacked the presence of necrosis and inflammatory cells surrounding the channels, were identified as VM (Figure 1A).

Of the 201 total HCC samples, LOXL2 cytoplasmic (LOXL2‐c) expression was detected in 112(55.7%), and LOXL2 nuclear (LOXL2‐n) expression was detected in 106 (52.7%) (Figure 1B). Of the 201 cases analysed, 48 were VM^+^ and 153 were VM^−^ (Table S1). In the VM^+^ group (n = 48), LOX2 expression included LOXL2‐c: 34 (70.8%) and LOXL2‐n: 30 (62.5%). In the VM^−^ group (n = 153), LOXL2 expression included LOXL2‐c: 77 (50.3%) and LOXL2‐n: 76 (49.7%). When comparing the VM^+^/VM^−^ and LOXL2 expression (Table S2), we found that LOXL2‐c expression differed significantly between the VM^+^ and VM^−^ groups (*P* = 0.002) but that LOXL2‐n expression did not. Furthermore, we found that LOXL2‐c expression was positively correlated with VM in the HCC samples by Spearman analysis; however, the LOXL2‐n expression was not.

By co‐staining of LOXL2 and VM, we found that the tissue which had VM formation, usually expressed high level of LOXL2‐c, while, the tissue without VM formation, had low expression of LOXL2‐c (Figure 1A). The analysis of the relationship between LOXL2 and the clinicopathological characteristics showed that the cytoplasmic LOXL2 overexpression was not correlated with the patients’ age or tumour size but was correlated with clinical metastasis (*P* = 0.002; and tumour grade (*P* = 0.04) (Table 1).

**Table 1:** Relationship between clinicopathological variables and LOXL2 expression

	Cases (n)	LOXL2 nuclei expression			LOXL2 cytoplasmic expression		
		+	−	*P*	+	−	*P*
Age (years)							
<50	59	30	29	0.758	33	26	0.537
≥50	142	76	66		72	70	
Tumour size							
T1: <5 cm	81	42	39	0.886	39	42	0.388
T2: ≥5 cm	120	64	56		66	54	
Grade							
I, II	96	45	51	0.001^a^	51	45	0.010^a^
III, IV	105	61	44		54	51	
Metastasis							
+	123	72	51	0.043^a^	75	48	0.002^a^
−	78	34	44		30	48	

a Statistically significant.

To validate the prediction value of LOXL2‐c expression for metastasis, discriminant analysis was performed to establish some predictive formulas from 160 HCC samples. By Pearson chi‐squared test, we compared differences between the predicted and actual metastasis in a second set of HCC cases, and found that LOXL2‐c expression had a good prediction value for HCC metastasis (Table S3).

The results of the Kaplan–Meier survival analysis showed that patients with VM and LOXL2‐c expression had a shorter survival period than those without VM or LOXL2‐c expression (Figure 1C), and that patients with LOXL2‐n expression did not show a short survival period than those without LOXL2‐n expression (Figure S1). LOXL2‐c overexpression and VM were independent predictors of HCC in the Cox analysis (Tables S7 and S8), while LOXL2‐n overexpression was not.

Together, these results indicated that LOXL2‐c expression is significantly associated with VM, metastasis, tumour grade and a shorter survival of HCC patients.

### 3.2 LOXL2 overexpression increased HCC cell invasion and migration and promoted the formation of tube‐like structures by HCC cells in vitro

**Figure 2:**
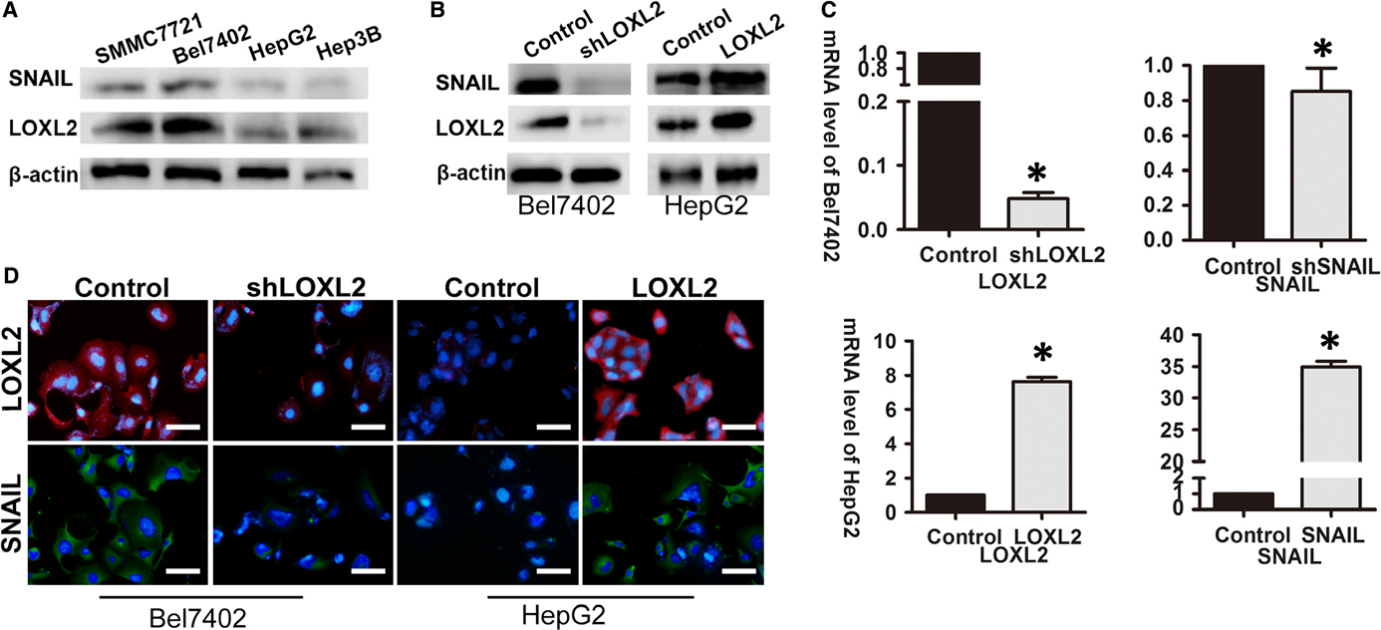
LOXL2 overexpression increased SNAIL expression in HCC cell in vitro. (A) LOXL2 and SNAIL expression was studied in liver cancer cell lines by western blot assay. LOXL2 expression level was higher in mesenchymal cells (Bel7402 and SMMC7721) compared to cell lines with a basal epithelial phenotype (HepG2 and Hep3B). (B) Western blot assay was used to detect the protein expression levels of LOXL2 and SNAIL in HepG‐Control cell and HepG2‐LOXL2 cell, Bel7402‐Control cell and Bel7402‐shLOXL2 cell. LOXL2 and SNAIL are upregulation in HepG2‐LOXL2 cell comparing with the HepG2‐control cell line. While in Bel7402‐shLOXL2 cell, LOXL2 is downregulation, comparing with Bel7402‐control cell line. (C) The mRNA expression level of LOXL2 and SNAIL in transfection cells was studied by qRT‐PCR. The mRNA expression level of LOXL2 has been upregulated in HepG2‐LOXL2 cell; and has been downregulated in Bel7402‐shLOXL2 cell. While The mRNA expression level of SNAIL has been upregulated in HepG2‐SNAIL cell; and has been downregulated in Bel7402‐shSNAIL cell. Error bars represent SD and *P < 0.05. (D) Efficiency of transfection was further confirmed by immunofluorescence analyses. LOXL2 is upregulation in HepG2‐LOXL2 cell comparing with the HepG2‐control cell line. And LOXL2 is downregulation in Bel7402‐shLOXL2 cell. On the other hand, SNAIL has been confirmed that it is upregulated in HepG2‐SNAIL cell; and downregulated in Bel7402‐shSNAIL cell. Scale bar represents 50 μm. Original magnification: 200×

In this section, we investigated the influence of LOXL2 on VM and metastasis in the HCC cell lines. First, the expression levels of LOXL2 were analysed in various HCC cell lines by western blot assay (Figure 2A). The epithelial HCC cells, such as HepG2 cells, exhibited lower LOXL2 expression, whereas the HCC cells with a mesenchymal phenotype, such as Bel7402 cells, exhibited higher LOXL2 expression (Figure 2A).

Thus, the above HCC cell lines were selected and transfected as recipient cells: HepG2 cells were transfected with the LOXL2 plasmid, and Bel7402 cells were transfected with the LOXL2 shRNA. Stable cell lines that over‐expressed or downregulated LOXL2 were established and tentatively designated as HepG2‐LOXL2 and Bel7402‐shLOXL2 cells (Figure 2B). The expression of LOXL2 in these cells was confirmed by western blot assay, immunofluorescence analyses and qRT‐PCR (Figure 2B‐D).

**Figure 3:**
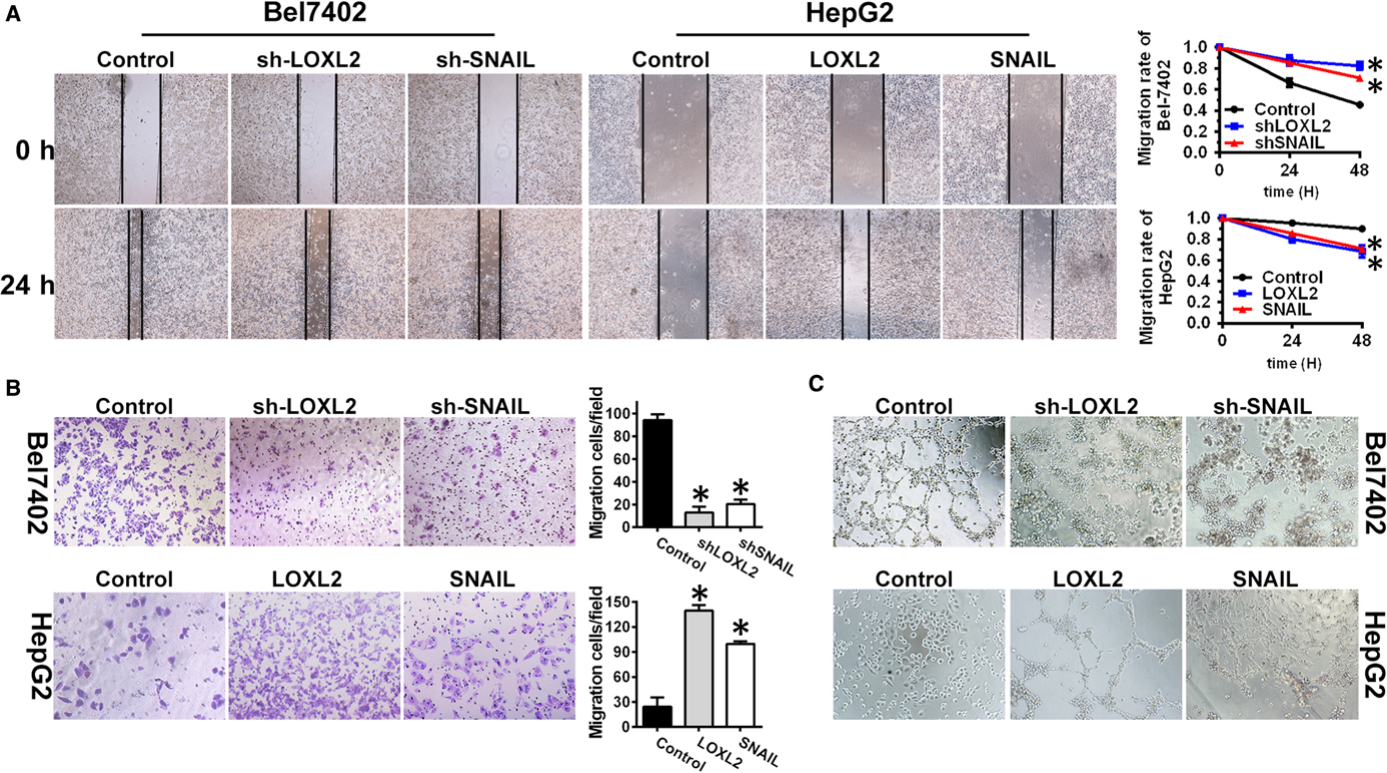
LOXL2 overexpression increased HCC cell invasion and migration, and promoted the formation of tube‐like structures by interacting with SNAIL in vitro. (A) The migratory properties among the Bel7402‐control, Bel7402‐shLOXL2 and Bel7402‐shSNAIL were analysed in wound healing assay; The migratory properties among the HepG2‐control, HepG2‐LOXL2 and HepG2‐SNAIL were analysed in wound healing assay (left). Migrated cells were plotted as the average number of cells per field of view from three different experiments (right), as described in the Section 2. (B) The invasion properties among the Bel7402‐control, Bel7402‐shLOXL2 and Bel7402‐shSNAIL, were analysed in transwell invasion assay; The migratory properties among the HepG2‐control, HepG2‐LOXL2 and HepG2‐SNAIL, were analysed in transwell invasion assay (left). Migrated cells were plotted as the average number of cells per field of view from three different experiments (right), as described in the Section 2. (C) The 3D VM formation assay was performed to analyse the abilities to form the vascular‐like tube in HCC cells. Error bars represent SD and *P < 0.05

Previous studies have shown that VM was associated with cell migration and invasion.^35^, ^36^ Therefore, the migration and invasion potential of the HepG2‐LOXL2 and Bel7402‐shLOXL2 cells were examined in vitro using a wound healing assay and Transwell migration assay. The results indicated that the stable overexpression of LOXL2 significantly increased the migration and invasion abilities of the HepG2 cells in vitro. Moreover, a significant decrease in cell migration and invasion was observed following the silencing of LOXL2 in the Bel7402‐shLOXL2 cells (Figure 3A,B). These data indicate that LOXL2 overexpression in HCC cells increased their migration and invasion abilities.

Next, to examine the influence of LOXL2 on VM, three‐dimensional cultures were used, and the results showed that VM was significantly increased in the HepG2‐LOXL2 cells compared with the control cells (*P* < 0.05) (Figure 3C); however, in the Bel7402‐shLOXL2 cells, VM was significantly suppressed by LOXL2 downregulation (*P* < 0.05) (Figure 3C). These results indicate that LOXL2 overexpression may increase the migration and invasion of HCC cells, thereby increasing VM in vitro, which provided further support for the possible role of LOXL2 in promoting VM formation.

Recently, Peinado H's research revealed that LOXL2 interact and cooperate with Snail to downregulate E‐cadherin expression, then induce an EMT progress.^29^ Also, in our study, we found that there is a positive association between LOXL2 and SNAIL. Giving that SNAIL is a very key factor of LOXL2, we have also studied the relationship between SNAIL expression and LOXL2 expression in vitro. The result showed that when LOXL2 expression level is higher in HepG2‐LOXL2 cells, comparing with control cells, the SNAIL expression level is higher. While LOXL2 expression level is lower in Bel7402‐shLOXL2 cells, comparing with control cells, the SNAIL expression level is lower (Figure 3A,B). This finding also gives us a clue to take our mechanism research.

### 3.3 LOXL2 overexpression promoted tumour growth, metastasis and VM formation in vivo

**Figure 4:**
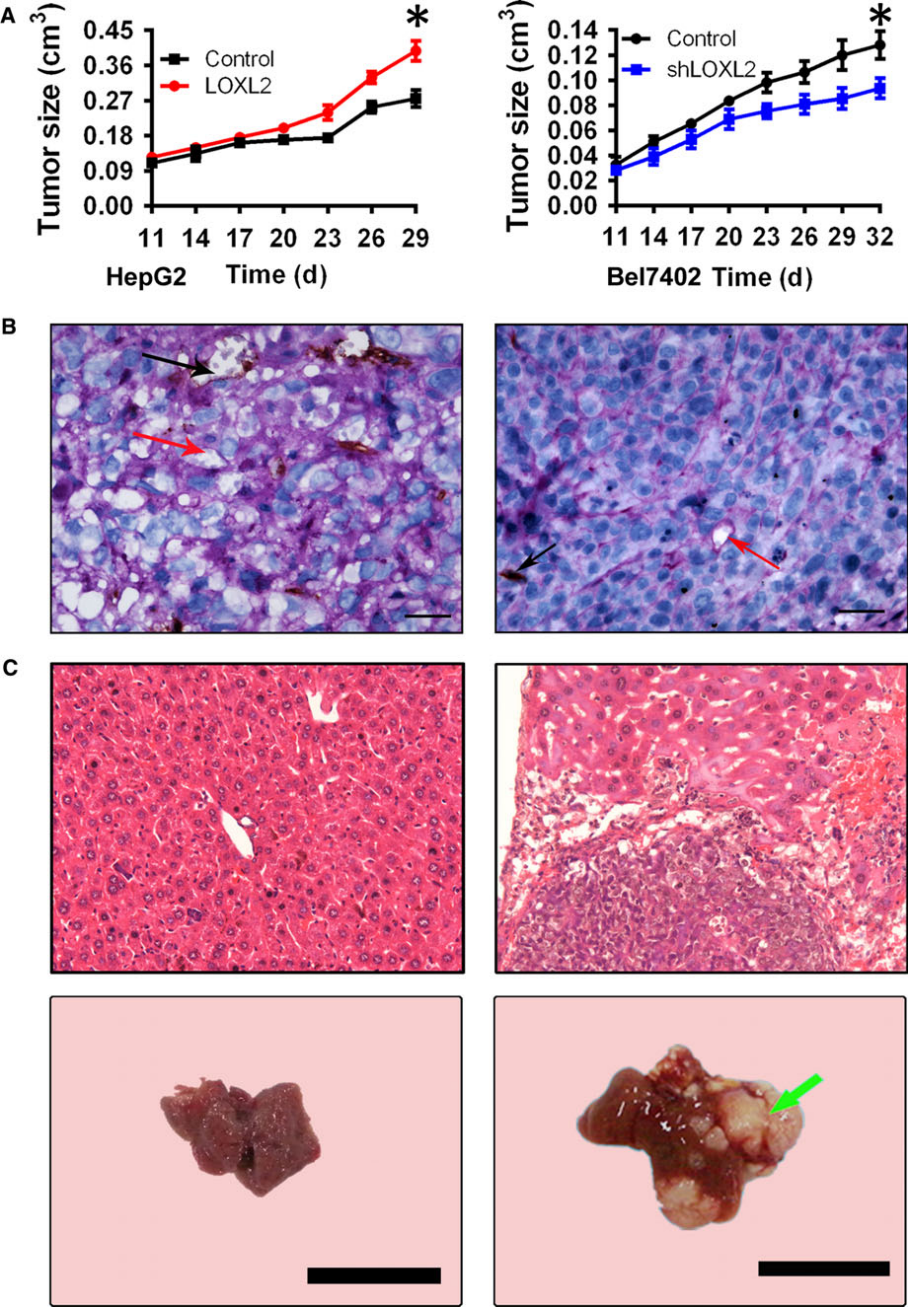
LOXL2 overexpression promoted tumour growth and VM formation in vivo. (A) HepG2 cells (stably transfected with LOXL2 or their control vectors) and Bel7402 cells (stably transfected with shLOXL2 or their control vectors) were subcutaneously injected into each mouse. As showed in the panel A, the xenografts showed a higher rate of tumour growth in HepG2‐LOXL2 as compared with the parental HepG2‐control cells; while a lower rate of tumour growth was showed in Bel7402‐shLOXL2 as compared with the parental Bel7402‐control cells. (B) Endomucin/PAS double staining showed VM in the HCC tissue (Red arrows indicate the capillary tubules formed by the tumour cells; Black arrows indicate the endothelium‐dependent vessels). Scale bar represents 50 μm. Original magnification: 200×. Error bars represent SD and *P < 0.05. (C) The HE staining in the xenografts showed that the migration rate in Bel7402‐control group is 22%, which is higher than the migration rate (0%) in Bel7402‐shLOXL2 group, (Green arrows indicate the metastatic tumours formed in a nude mouse xenograft model)

We further investigated the effects of LOXL2 effects on tumourigenic potential, growth, migration and progression in vivo using a subcutaneous xenograft tumour model. The tumours induced by the HepG2‐LOXL2 cells formed primary tumours at all injection sites (eight tumours/eight injection sites) at 7 days after injection, similar to the control group. The tumours induced by the HepG2‐LOXL2 cells also showed a significant higher growth rate than those induced by the HepG2‐control cells at 23 and 27 days after injection (Figure 4A). Compared with the control cells, a 40% increase in the volume of the tumours induced by HepG2‐LOXL2 cells was detected at 32 days after inoculation. The Bel7402‐shLOXL2 tumours emerged at 7 days after injection. Fewer tumours (87%) were observed in the flanks injected with Bel7402‐shLOXL2 cells compared with the tumours that appeared at the sites injected with Bel7402‐control transfectants in 8 of the 8 (100%) mice. Moreover, the tumours induced by Bel7402‐shLOXL2 cells grew at much slower rate than did those induced by the Bel7402‐control cells (Figure 4A). Indeed, 37 days after injection, a 33% reduction in the volume of tumours induced by the Bel7402‐shLOXL2 cells was observed. Although the growth conditions were different for the HepG2 and Bel7402 groups, LOXL2 promoted tumour growth in both the HepG2‐LOXL2 cell‐engrafted tumours and Bel7402‐control cell‐engrafted tumours. These results indicate that the introduction of LOXL2 significantly promoted tumourigenicity and tumour growth in a nude mouse xenograft model.

Vasculogenic mimicry was identified by endomucin/PAS double staining. The results showed that VM exists in tumours induced by the HepG2‐LOXL2 cells and in those induced by the Bel7402‐control cells (Figure 4B). What's more, the metastasis array results showed that the migration rate in Bel7402‐control group is 22%, which is higher than the migration rate (0%) in Bel7402‐shLOXL2 group (Figure 4C). Taken together, these results strongly suggest that LOXL2 is required for the efficient growth, VM formation, metastasis and progression of malignant tumours.

### 3.4 LOXL2 expression promoted VM by collaborating with SNAIL

Recently, the research revealed that the collaboration of LOXL2 with SNAIL and LLGL2 influences EMT and cell polarity respectively.^20^, ^22^, ^23^, ^24^ To gain insight into the mechanism by which LOXL2 promotes VM, we studied the expression of LOXL2‐associated factors and the relationships among them in HCC tissue samples, HCC cell lines and the xenograft model.

**Figure 5:**
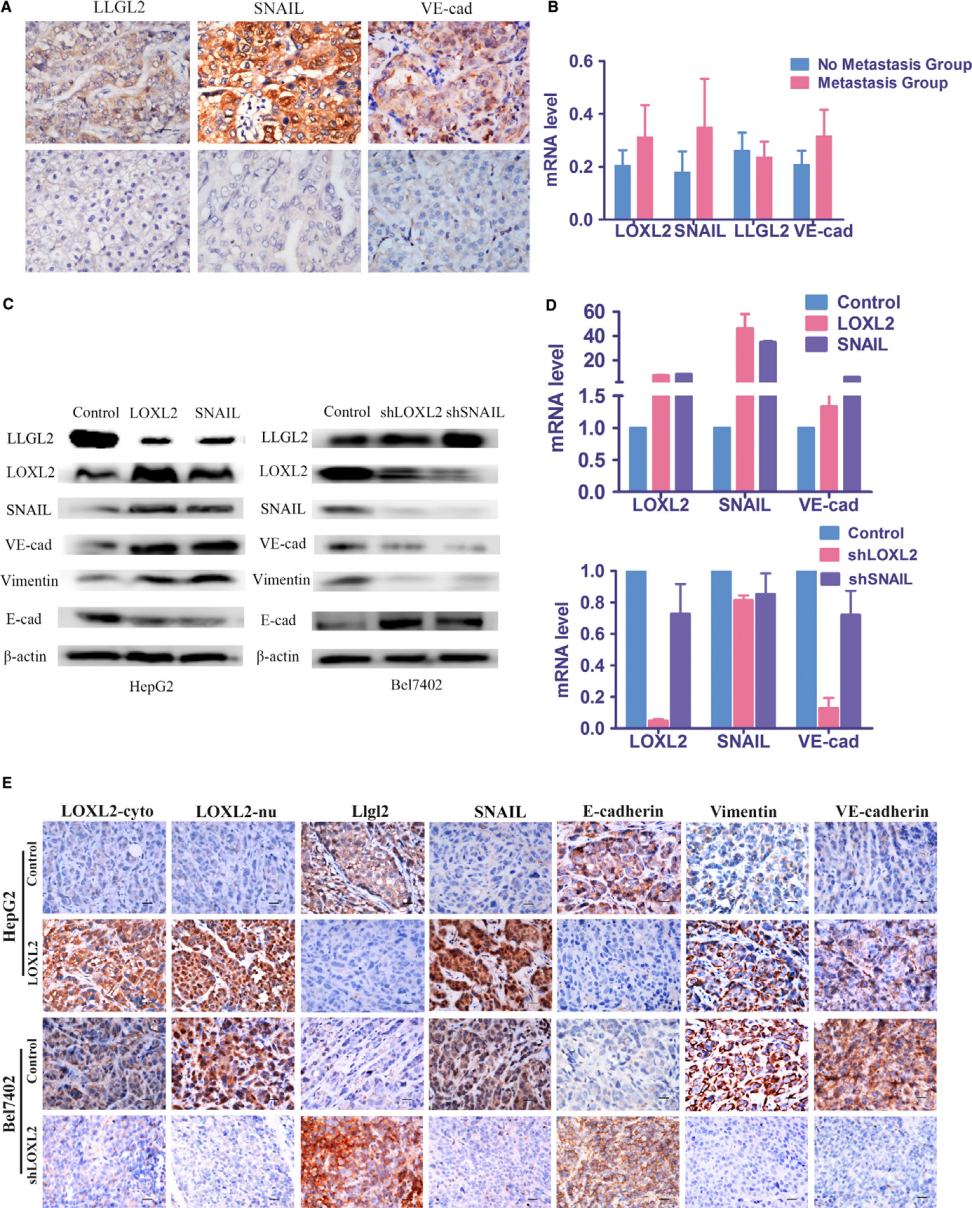
LOXL2 expression promoted VM by collaborating with SNAIL. (A) LOXL2, LLGL2, VE‐cadherin (VE‐cad) and SNAIL expression and their association with metastasis in Paraffin‐embedded tissues. The upper panel is the positive staining of LLGL2, SNAIL and VE‐cadherin in human HCC samples, while the lower panel is negative control. (B) LOXL2, LLGL2, VE‐cadherin (VE‐cad) and SNAIL expression and their association with metastasis in frozen HCC tissues. GAPDH was used as a loading control for qRT‐PCR. (C) Expression of E‐cadherin, vimentin, SNAIL, VE‐cadherin and LLGL2 in LOXL2 stably transfected HepG2 cells and shLOXL2 stably transfected Bel7402 cells were detected by western blot assay. Expression of E‐cadherin, vimentin, LOXL2, VE‐cadherin and LLGL2 in SNAIL stably transfected HepG2 cells and shSNAIL stably transfected Bel7402 cells were detected by western blot assay. β‐actin was used as a loading control. (D) The mRNA level of LLGL2 and VE‐cadherin in HCC cell was detected by qRT‐PCR. SNAIL and VE‐cadherin mRNA expression was upregulated in HepG2‐LOXL2 cell, comparing with HepG2‐control. LOXL2 and VE‐cadherin mRNA expression was upregulated in HepG2‐SNAIL cell, comparing with HepG2‐control. Among the Bel7402‐shLOXL2 cell, SNAIL and VE‐cadherin mRNA expression was downregulated, comparing with Bel7402‐control. In Bel7402‐shSNAIL cell, LOXL2 and VE‐cadherin mRNA expression was upregulated, comparing with Bel7402‐control. GAPDH was used as a loading control for qRT‐PCR. (E) HepG2 cells stably transfected with LOXL2 and Bel7402 cells stably transfected with shLOXL2 were subcutaneously injected into BALB/c‐nu/nu mice. IHC staining showed the expression of LOXL2, E‐cadherin, VE‐cadherin, vimentin, SNAIL and LLGL2 in the groups injected with the HepG2‐LOXL2 cells and Bel7402 cells transfected with shLOXL2. Error bars represent SD and *P < 0.05. Scale bar represents 50 μm. Original magnification: 200×

In the 201 paraffin‐embedded HCC samples, IHC analysis was performed to assess the expression of SNAIL and LLGL2 and the VM‐related protein VE‐cadherin (Figure 5A). The expression of SNAIL and VE‐cadherin was significantly high in the HCC samples, whereas the expression of LLGL2 was low. Using the Pearson correlation test, we found that there were significant associations between LOXL2‐c, VE‐cadherin and SNAIL expression and VM (Table S5). LOXL2 expression was positively correlated with VE‐cadherin and SNAIL expression and VM and was inversely correlated with LLGL2 expression. SNAIL expression was positively correlated with VE‐cadherin expression and VM and was inversely correlated with LLGL2 expression. VE‐cadherin expression was positively correlated with VM and was inversely correlated with LLGL2 expression. LLGL2 expression was inversely correlated with VM.

The expression levels of LOXL2, SNAIL, LLGL2 and VE‐cadherin were further validated by qRT‐PCR in 31 HCC tissues and 33 normal liver tissues (Figure 5B). Notably, the results showed that the expression of LOXL2, SNAIL and VE‐cadherin was significantly increased in the HCC tissues compared with the non‐cancerous tissues. Conversely, LLGL2 expression was reduced in the HCC tissues compared with the noncancerous tissues (Figure 5B, *P* < 0.05).

The above findings indicated that LOXL2 overexpression may promote the expression of SNAIL and VE‐cadherin. It is believed that VE‐cadherin, a critical marker in tumour VM, is usually present in tumours with VM. Given the relationship between LOXL2, SNAIL, VE‐cadherin and LLGL2 expression and VM, we proposed that LOXL2 may promote VM by increasing SNAIL expression.

Alternatively, Bel7402 cells, which display high endogenous SNAIL expression, were transfected with a shSNAIL‐RNA; Bel7402 cells transfected with a control vector served as the control treatment. HepG2 cells, which expressed low levels of SNAIL, were transfected with the LOXL2 plasmid, and HepG2 cells transfected with a control vector served as the control treatment (Figure 5C).

In vitro, western blot assay and qRT‐PCR were firstly used to analyse the protein and mRNA levels of LOXL2, SNAIL, VE‐cadherin, LLGL2, the epithelial marker E‐cadherin and the mesenchymal marker vimentin following the upregulation and downregulation of LOXL2 and SNAIL expression (Figure 5C,D). HepG2‐LOXL2 cells expressed high levels of SNAIL, VE‐cadherin and vimentin and low levels of LLGL2 and E‐cadherin compared with the HepG2‐control cells. In contrast, the Bel7402‐shLOXL2 cells expressed low levels of SNAIL, VE‐cadherin and vimentin and high levels of LLGL2 and E‐cadherin compared with the Bel7402‐control cells.

Then, changes in the protein expression levels and cellular behaviours were evaluated to verify the role of SNAIL in the progress by which LOXL2 overexpression promote VM. The western blot assay results showed that in the HepG2‐SNAIL cells, LOXL2, VE‐cadherin and vimentin were expressed at high levels and LLGL2 and E‐cadherin were expressed at low levels; the opposite results were observed for Bel7402‐shSNAIL cells (Figure 5C).

We assessed the role of SNAIL in regulating HCC cell migration and invasion using wound healing assays and transwell assays in Bel7402 and HepG2 cells. These results indicated that SNAIL overexpression promoted cell migration and invasion, similar to LOXL2 overexpression in HepG2 cells. Conversely, reduced SNAIL expression repressed cell migration and invasion, similar to shLOXL2 in the Bel7402 cells (Figure 3A,B).

To explore the biological significance of SNAIL in VM, a well‐established in vitro model of VM (3D culture) was used. The results showed that SNAIL significantly promoted capillary tube formation by cultures of Bel7402 and HepG2 cells within the 3D matrigel medium, and this effect of SNAIL was consistent with that of LOXL2 (Figure 3C). These data indicated that SNAIL overexpression exerts a similar effect as LOXL2 overexpression's effect on cell migration and invasion and capillary tube formation.

In the mouse tumour tissues, IHC analysis revealed that increased expression of SNAIL, VE‐cadherin and vimentin was observed in the HepG2‐LOXL2 cell‐xenograft tumours compared with the HepG2‐control cell‐xenograft tumours, whereas LLGL2 and E‐cadherin were expressed at lower levels. The opposite results were detected in the Bel7402‐shLOXL2 cell‐xenograft tumours. These results suggested that the tumours induced by LOXL2 overexpression showed a high level of SNAIL, VE‐cadherin and vimentin and low level of LLGL2 and E‐cadherin, and played an important role in contributing to VM in vivo (Figure 5D). Therefore, we propose that LOXL2 may increase SNAIL expression, thereby enabling VM.

All of these data from the HCC tissue samples, HCC cell lines and xenograft model preliminarily verify the mechanism by which LOXL2 promotes VM.

## 4 DISCUSSION

Tumour growth and invasion are dependent on a persistent blood supply.^5^ Here, we showed that LOXL2 overexpression promotes VM, which is a form of angiogenesis, and has a good predictive value for HCC metastasis and prognosis; thus, these results can provide deeper insights into tumour progression.

Vasculogenic mimicry has increasingly been considered to function simultaneously with angiogenesis.^9^, ^37^ A recent study revealed that LOXL2 regulates capillary formation and is required for tumour angiogenesis.^25^, ^26^ Consistently, in our study, we have reported that LOXL2 promotes VM in HCC. VM channels occur in at least 10 tumour types that are aggressive, highly metastatic and inclined to poor differentiation.^6^, ^10^, ^13^, ^14^ The term VM refers to the ability of aggressive tumour cells to form periodic acid‐Schiff‐positive and CD31‐negative cells that line VM networks in vivo and form tubular structures and patterned networks in three‐dimensional (3D) cultures in vitro.^10^, ^12^

The results of the three‐dimensional cultures of HCC cells and endomucin/PAS double staining in the mouse tumour tissues showed that LOXL2 promoted VM.^6^, ^14^, ^38^, ^39^ This result agrees with other reports showing that LOXL2 is involved in angiogenesis. Due to the special function of VM, tumour cells could be exposed to blood flow, allowing them to enter the microcirculation and metastasize to other organs.^9^, ^27^ Thus, VM is an important factor in tumour progression that not only leads to a poor prognosis but also explains at least part of the poor targeting of endothelial cells by anti‐angiogenic treatments.^38^ Our previous research data have revealed that patients with VM^+^ tumours tend to have poorer outcomes than patients with VM^−^ tumours.^11^, ^15^ Furthermore, the presence of VM in highly invasive tumours is linked to a high tumour grade, invasiveness, metastasis and shorter survival.^15^ The relationships between LOXL2‐c expression and tumour size and grade were detected in our study. We found that LOXL2‐c expression was significantly correlated with tumour grade and metastasis, which is consistent with a previous study.^25^ The Kaplan–Meier survival analysis showed that there was a positive correlations between LOXL2‐c expression and VM with poor overall survival (OS) and disease free survival (DFS) (*P* < 0.05). Also, the Cox regression LOXL2‐c expression was an independent prognostic factor for OS and DFS, which is consistent with previous reports that LOXL2 promotes metastasis and is linked to poor prognosis in HCC patients. Although there was correlation between LOXL2 nuclear expression and tumour grade clinical metastasis and lower OS, the nuclear expression of LOXL2 have no significant difference. What's more, Moreno‐Bueno et al have reported that the cytoplasmic/perinuclear expression of LOXL2 associated with increased tumourigenicity and promoted metastatic potential.^28^

To further validate the predictive value of LOXL2‐c, we established a combination predictive formula using discriminant analysis. Using Pearson's chi‐squared test, we compared the differences between predicted and actual metastasis in a second set of HCC cases and found that this formula has a good predictive value for HCC metastasis. Consistent with our result, LOXL2 has been reported as a key determinant of HCC metastasis and a good diagnostic marker for HCC patients. Recent investigations have also indicated that LOXL2 is a predictor of poor prognosis in specific tumour types, such as larynx squamous cell carcinomas and is associated with metastatic dissemination in basal breast carcinomas.^28^, ^30^ Therefore, we may conclude that LOXL2‐c is a good predictor of a poor prognosis in HCC patients.

Our previous research has showed that tumour cells that form VM networks secrete matrix metalloproteinases and VE‐cadherin and laminin, which induce ECM remodelling and promote VM formation.^6^, ^14^, ^37^, ^38^, ^39^, ^40^ In this study, we found that LOXL2‐c overexpression was positively correlated with SNAIL and VE‐cadherin expression and VM and negatively correlated with LLGL2 expression in HCC tumour tissues, HCC cell lines and the xenograft model. These results indicate that LOXL2 may promote VM by promoting SNAIL and VE‐cadherin expression. Peinado et al have reported that LOXL2 can interact and cooperate with SNAIL to downregulate E‐cadherin expression.^29^ We proposed that LOXL2 may promote VM by collaborating with SNAIL.

As shown in a previous study, EMT regulators and VM are correlated, and many EMT regulators, such as Twist and Slug, can promote VM.^14^, ^33^, ^41^ Similarly, in the present study, we found that in another EMT regulator, the expression of SNAIL correlates with VM in HCC, and SNAIL itself exerts a positive effect on cell migration and invasion and capillary tube formation. Interestingly, we found that SNAIL can promote VM in HCC cell which is consistent to Sun D's research.^33^ All of these results indicated that LOXL2 promotes VM in HCC by collaborating with SNAIL. Our results also showed that LOXL2 positively correlated with VE‐cadherin expression and VM. VE‐cadherin is one of the first molecules that was identified as a VM promoter, and it is critical in VM.^14^, ^35^, ^42^, ^43^ Together, these results indicate that LOXL2 collaborated with SNAIL, further promoted the expression of the VM marker VE‐cadherin, and increased VM in HCC. In addition, the results that the LOXL2 protein and mRNA levels were negatively correlated with LLGL2 expression in HCC tumour tissues and HCC cell lines were detected in this study. Correspondingly, LLGL2 expression was negatively correlated with LOXL2, SNAIL, VE‐cadherin expression and VM. All these results consistent with the research that LOXL2 maintains the mesenchymal phenotype of carcinoma cells via a novel mechanism that involves the transcriptional downregulation of LLGL2 and disorganization of cell polarity and tight junction complexes^28^ This result may further explain the interaction between LOXL2 and SNAIL in the progress of VM. As it is shown in the experiments in vivo and vitro, LOXL2 can not only promote HCC migration and invasion in vitro but also promote the metastasis of HCC in vivo.

## 5 CONCLUSIONS

In conclusion, we have shown, for the first time, that there are positive correlations among LOXL2‐c and SNAIL expression and VM in HCC; moreover, our present results indicate that LOXL2 may upregulate the expression of SNAIL and VE‐cadherin, and promote VM, ultimately facilitating tumour metastasis and indicating a poor prognosis in HCC patients. Our findings not only provide new insights into the molecular mechanism of VM and metastasis in HCC but also suggest a novel therapeutic target for the inhibition of HCC metastasis.

## CONFLICT OF INTEREST

No conflicts of interest were disclosed.
