# Supplementary figures and images for: LOXL2 promotes vasculogenic mimicry and tumour aggressiveness in hepatocellular carcinoma
Source: J Cell Mol Med. 2018 Dec 1;23(2):1363–74. doi: 10.1111/jcmm.14039 (PMC6349148; doi:10.1111/jcmm.14039)

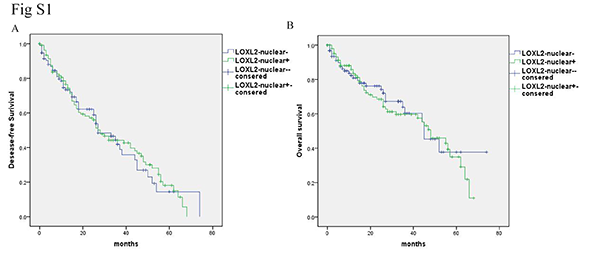

Supplement: Supplementary file 1 [file JCMM-23-1363-s001.tif]

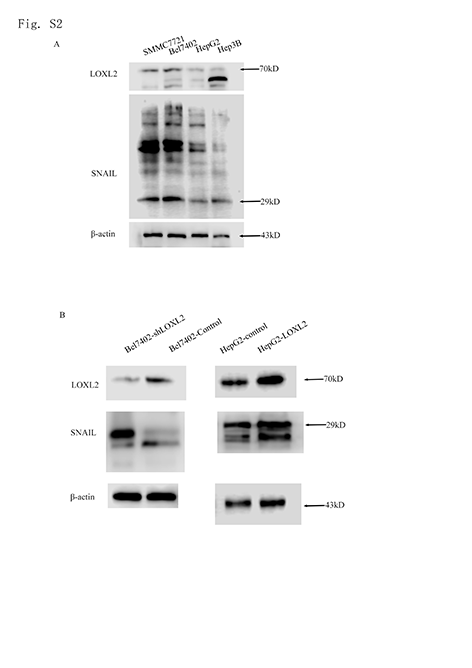

Supplement: Supplementary file 2 [file JCMM-23-1363-s002.tif]

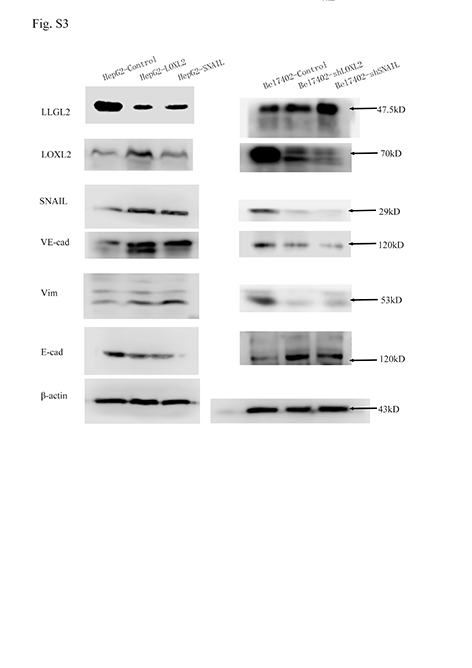

Supplement: Supplementary file 3 [file JCMM-23-1363-s003.tif]
