# Supplementary material for: LOXL2 promotes vasculogenic mimicry and tumour aggressiveness in hepatocellular carcinoma
Source: J Cell Mol Med. 2018 Dec 1;23(2):1363–74. doi: 10.1111/jcmm.14039 (PMC6349148; doi:10.1111/jcmm.14039)
Supplement: Supplementary file 4 [file JCMM-23-1363-s004.doc]

**LOXL2 promotes vasculogenic mimicry and tumor aggressiveness in hepatocellular carcinoma**

Bing Shao^2,3^,Xiulan Zhao^1,2*^, Tieju Liu^1,2^,Yanhui Zhang^3^,Ran Sun^4^, Xueyi Dong^1,2^,Fang Liu^1,2^,Nan Zhao^1,2^,Danfang Zhang^1,2^, Lili Wu^2^,Yong Wang^2^, Meili Wang^2^, Jie Meng^1,2^, Xian Lin^2^, Baocun Sun^1,2,3*^

1 Department of Pathology, General Hospital of Tianjin Medical University, Tianjin 300052, China

2 Department of Pathology, Tianjin Medical University, Tianjin 300070, China

3 Department of Pathology, Cancer Hospital of Tianjin Medical University, Tianjin 300060, China

4 Tianjin Nankai Hospital, Tianjin 300100, China

**Running Title**: LOXL2 in HCC with VM formation

**Keywords:** cell polarity protein, tumor metastasis, angiogenesis, tumor progression, prognosis

**Corresponding author:** Xiulan Zhao, Baocun Sun

General Hospital and Department of Pathology and Cancer Hospital of Tianjin Medical University, Tianjin 300052,PR China;

Tel: 86-13602042200.

Fax: 86-22-83336813.

E-mail: [xiulanzhao@aliyun.com](mailto:xiulanzhao@aliyun.com)；

**Supplementary Data**

**Table s1.The expression of LOXL2**


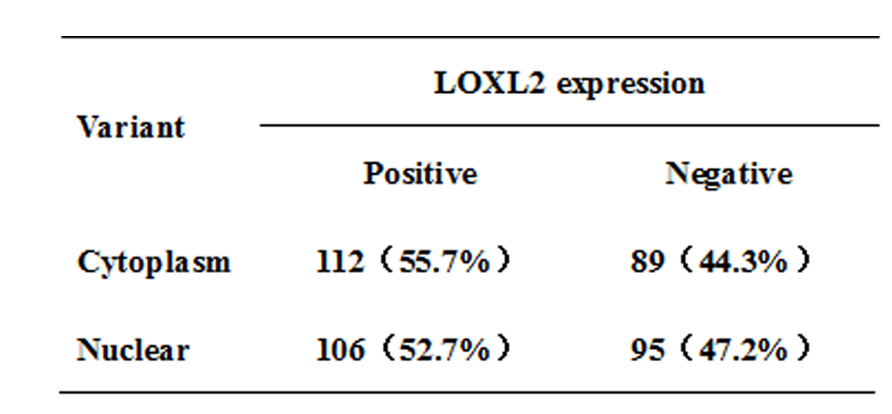


**Table s2. The correlation between LOXL2 and VM formation**


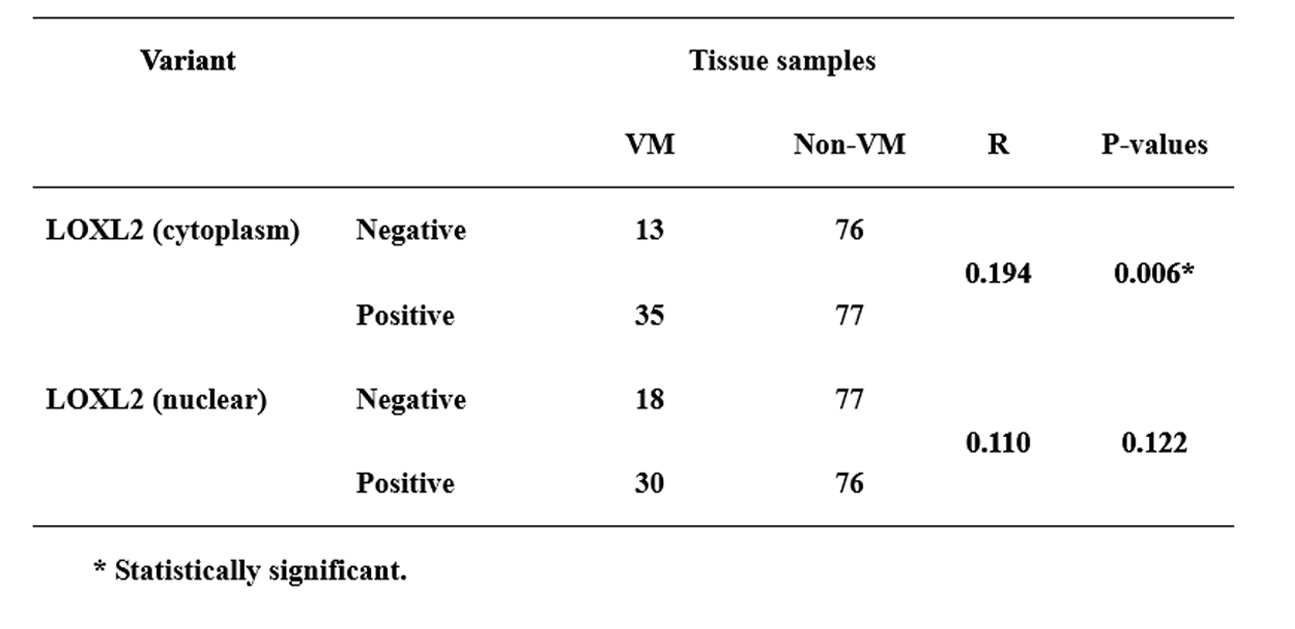


**Table s3. Prediction values of LOXL2 and SNAIL in HCC.**

|  | Prediction formula | Se (%) | FN (%) | Sp (%) | FP (%) | Agreement rate (%) | PV+ (%) | PV− (%) | P |
| --- | --- | --- | --- | --- | --- | --- | --- | --- | --- |
| Combination | D=1.015×LOXL2 + 1.038×SNAIL − 1.638 | 68.3 | 33.3 | 59.8 | 40.5 | 25.4 | 54.5 | 71.0 | 0.047 |
| LOXL2 | D=2.042×LOXL2 – 1.067 | 58.1 | 47.6 | 58.3 | 40.9 | 10.3 | 39.4 | 71.0 | 0.438 |
| SNAIL | D=2.206×SNAIL − 1.913 | 63.4 | 57.6 | 56.2 | 36.4 | 16.4 | 42.4 | 74.2 | 0.195 |

FN: false-negative rate; FP: false-positive rate; PV−: negative predictive value; PV+: positive predictive value; Se: sensitivity; Sp: specificity

Cases are considered potentially metastatic if D ≥ 1; and not potentially metastatic if D is near to 0 or < 0.

**Table s4 The information of antibodies used in this study**

| Antibodies | Company | Company | Product Number | Pretreatment | Dilution  IHC | Positive location |
| --- | --- | --- | --- | --- | --- | --- |
| LOXL2 | Gene Tex | Rabbit | GTX105085 | Microwave | 1:800 | Nuclear and/or Cytoplasmic |
| LLGL2 | Abcam | Mouse | AB-55423 | Microwave | 1:300 | Cytoplasmic |
| SNAIL | Gene Tex | Rabbit | GTX125918 | Microwave | 1:25 | Nuclear and/or Cytoplasmic |
| VE-Cad | Abcam | Rabbit | AB-33168 | Microwave | 1:800 | Membranous  and/or Cytoplasmic |
| E-cad | Santa Cruz | Rabbit | SC-7870 | Microwave | 1:100 | Membranous |
| Vimentin | ZSGB-BIO | Mouse | ZM0260 | Microwave | 1:400 | Cytoplasmic |
| CD31 | ZSGB-BIO | Mouse | ZM0044 | Microwave | 1:50 | Cytoplasmic |
| Endomucin | eBioscience | Rat | 14-5851-81 | Microwave | 1:600 | Membranous |

**Table s5. Correlation between LOXL2,LLGL2,**

**SNAIL and VE-cadherin.** (shown r value)

|  | LOXL2 (cytoplasm) | LOXL2 (nuclear) | LLGL2 | SNAIL | VE-Cad |
| --- | --- | --- | --- | --- | --- |
| LOXL2 (cytoplasm) | NS | 0.148 | -0.110 | 0.165 | 0.235 |
| LOXL2 (nuclear) | — | NS | -0.210 | 0.152 | NS |
| LLGL2 | — | — | NS | NS | NS |
| SNAIL | — | — | — | NS | NS |
| VE-Cad | — | — | — | — | NS |

NS: *p*>0.05

**Table s6. Relationships between metastasis and expr**ession of LLGL2, VE-cadherin and SNAIL expression in hepatocellular carcinoma (HCC) (χ2 test).

| Variant |  | Tissue samples χ2 test | | P-value |
| --- | --- | --- | --- | --- |
|  |  | Metastasis+ | Metastasis− | P |
| LLGL2 | Negative | 71 | 56 | 0.051 |
|  | Positive | 52 | 22 |  |
| VE-cadherin | Negative | 100 | 57 | 0.265 |
|  | Positive | 23 | 21 |  |
| SNAIL | Negative | 21 | 27 | 0.006* |
|  | Positive | 102 | 51 |  |
| VM | Negative | 76 | 77 | 0.000* |
|  | Positive | 47 | 1 |  |

*Statistically significant.

**Table s7**. Cox proportional-hazard regression model analysis for overall survival.

|  | HR** | 95%CI | P |
| --- | --- | --- | --- |
| Age (years) |  |  |  |
| <50, ≥50 | 1.846 | 1.161–2.936 | 0.063 |
| Tumor size |  |  |  |
| T1, T2 | 6.308 | 3.602–11.046 | 0.070 |
| Grade |  |  |  |
| I,II,III,IV | 2.844 | 1.724–4.691 | 0.092 |
| VM |  |  |  |
| Positive, negative | 0.691 | 0.428–1.116 | 0.005* |
| LOXL2,cytoplasm |  |  |  |
| Positive, negative | 1.564 | 1.060–2.306 | 0.024* |
| LOXL2,nuclei |  |  |  |
| Positive, negative | 0.739 | 0.494–1.104 | 0.140 |
| LLGL2 |  |  |  |
| Positive, negative | 1.947 | 1.315–2.883 | 0.101 |
| SNAIL |  |  |  |
| Positive, negative | 0.792 | 0.466–1.346 | 0.389 |
| VE-cad |  |  |  |
| Positive, negative | 1.121 | 0.776–1.619 | 0.554 |

* Statistically significant.

**Table s8** Cox proportional-hazard regression model analysis for disease-free survival.

|  | HR** | 95%CI | *P* |
| --- | --- | --- | --- |
| Age (years) |  |  |  |
| <50, ≥50 | 2.312 | 1.276 –4.192 | 0.096 |
| Tumor size |  |  |  |
| T1, T2 | 8.963 | 4.416 –18.193 | 0.104 |
| Grade |  |  |  |
| I, II, III, IV | 2.854 | 1.500–5.429 | 1.322 |
| VM |  |  |  |
| Positive, Negative | 1.185 | 0.654 –2.148 | 0.005* |
| LOXL2,cytoplasmic |  |  |  |
| Positive, negative | 2.156 | 1.256–3.699 | 0.005* |
| LOXL2,nuclei |  |  |  |
| Positive, negative | 0.761 | 0.456–1.271 | 0.297 |
| LLGL2 |  |  |  |
| Positive, negative | 2.447 | 1.475–4.060 | 0.001* |
| SNAIL |  |  |  |
| Positive, negative | 0.792 | 0.466–1.346 | 0.389 |
| VE-cad |  |  |  |
| Positive, negative | 0.988 | 0.480–2.034 | 0.973 |

*Statistically significant.

**Figure legend**

**Figure S1:** **Patients with LOXL2- n expression did not had a shorter survival period than those without LOXL2- n expression**

(A) Patients with LOXL2- n expression did not had a shorter OS survival period than those without LOXL2- n expression;

(B) Patients with LOXL2- n expression did not had a shorter DFS survival period than those without LOXL2- n expression.

**Figure S2:** **The expression of LOXL2 and SNAIL in liver cancer cell lines and transfected cells** **was confirmed by western blotting.**

(A) LOXL2 and SNAIL expression was studied in liver cancer cell lines by western blotting. LOXL2 expression level was higher in mesenchymal cells (Bel7402 and SMMC7721) compared to cell lines with a basal epithelial phenotype (HepG2 and Hep3B), which indicated that LOXL2 was associated with the EMT phenotype in HCC cells.

(B) The expression of LOXL2 and SNAIL in transfected cells was confirmed by western blotting

**Figure S3:** **Expression of E-cadherin, vimentin, SNAIL, VE-cadherin and LLGL2 in stably transfected cells was detected by Western blotting**

**Supplement material of Materials and methods**

**Counting methods**

The protein expression levels were quantified as previously described ([28](#_ENREF_37),. Both the intensity and the percentage of positive cells were evaluated. In each slide, we determined the staining intensity as 0: no stain; 1: weakly positive (faint yellow); 2: moderately positive; or 3: strongly positive (brown staining). To determine the percentages of positive cells, we observed at least 10 randomly chosen fields in each section under a microscope, and counted 100 tumor cells in each field. The number of positive cells was counted and the cell expression levels were stratified as <10% positive cells: 0 (negative); 10–25%: 1 (weak); 25–50%: 2 (moderate); and >50%: 3 (strong). The product of the staining intensity and positive cell scores was the final score for each section.

**Murine Xenograft Model**

Five-week-old female BALB/c-null mice were housed in the animal facilities of the Tianjin Medical University, and the study was approved by the Institutional Animal Care and Use Committee. HepG2 cells and Bel7402 cells (10^7^ cells/ml), which were stably transfected with LOXL2,shLOXL2, and their control vectors, were mixed with Matrigel (BD Bioscience) and subcutaneously injected into the upper right flanks of nude mice (0.1 mL/mouse). The mice were monitored for 4-5 weeks and tumor sizes were measured daily using a caliper. The experiments were terminated because the HepG2-LOXL2/Control and Bel7402-shLOXL2/Control cells tended to become necrotic and form skin ulcers. After the observations were complete, the mice were sacrificed. Tumors were harvested and stored at -80°C for the subsequent tests, and some of them was fixed with 10% formaldehyde for histological examination.

To evaluate the influence of LOXL2 on the migration of hepatocellular carcinoma in vivo. We performed the metastasis assays in 4-6 week-old nude mice. For each mouse, 5×10^6^ hepatocellular carcinoma cell was injected in the upper pole of the spleen with a microsyringe under anesthesia. After 6 or 8 weeks mice were sacrificed and their spleens and livers were harvested and fixed with 10% formaldehyde, and then used for the sequent histological examination.
